# Supporting Caregivers Remotely During a Pandemic: Comparison of WHO Caregiver Skills Training Delivered Online Versus in Person in Public Health Settings in Italy

**DOI:** 10.1007/s10803-022-05800-y

**Published:** 2022-12-01

**Authors:** Camilla Ferrante, Paola Sorgato, Mariachiara Fioravanti, Laura Pacione, Giuseppe Maurizio Arduino, Sabrina Ghersi, Maria Luisa Scattoni, CST Italy Team, Camilla Chiesa, Donatella Elia, Elisabetta Gonella, Sara Rizzo, Arianna Salandin, WHO CST Team, Felicity L. Brown, Stephanie Shire, Chiara Servili, Erica Salomone

**Affiliations:** 1 Department of Psychology, University of Milan-Bicocca, Piazza dell’Ateneo Nuovo 1, 20126 Milan, Italy; 2 Department of Mental Health and Substance Use, World Health Organization, Geneva, Switzerland; 3 Centro Autismo e Sindrome di Asperger, Ospedale Regina Montis Regalis Mondovì, Mondovì (Cuneo), Italy; 4 Fondazione Paideia, Turin, Italy; 5 Istituto Superiore di Sanità, Rome, Italy

**Keywords:** Caregiver-mediated intervention, Telepractice, Virtual adaptation, Autism spectrum disorder, WHO Caregiver Skills Training

## Abstract

Feasibility, acceptability and effectiveness data of a virtual adaptation of the WHO Caregiver Skills Training (CST; n = 25) were compared with those of a pilot RCT of CST delivered in person (n = 43) against treatment as usual (TAU; n = 43). Virtual CST was delivered with high levels of integrity, but received lower ratings in some caregiver- and facilitator-rated acceptability and feasibility dimensions. Qualitative analysis identified both benefits (flexibility, convenience, clinical usefulness) and challenges, (technological issues, distraction from family members, emotional distance). Virtual and in-person CST improved significantly more on caregiver competence than TAU; there were no other significant effects. Potential for use of virtual CST as a clinical response in contexts where in-person delivery is not possible is discussed.

**Supplementary Information:**

The online version contains supplementary material available at 10.1007/s10803-022-05800-y.

## Introduction

### Potential of Telehealth to Address the Treatment Gap for ASD

Intervening early with evidence-based psychological therapies for children with autism spectrum disorder (ASD) is key to optimize development and wellbeing trajectories (Pierce et al., 2016). Despite this, access to early interventions is inadequate in low-and-middle-income countries (LMIC) (Reichow et al., 2013) as well as in high-income countries (HIC), including countries in the European region (Bejarano-Martín et al., 2020; Salomone et al., 2016) and the United States (Smith et al., 2020). This is linked to specific barriers that limit access to services, including cost of treatment for families (Elder et al., 2016) or insufficient number of trained health professionals (Elder et al., 2016; Esposito et al., 2020), large waiting lists, unavailability of specific services in non-metropolitan areas (Monz et al., 2019) and cost and time for travel to clinics (Elder et al., 2016; Murphy et al., 2021). To respond to this treatment gap, interventions adapted for delivery via telehealth have been implemented for a few decades (Reed et al., 2000), however not as mainstream options within public health systems. More recently, the COVID-19 emergency accelerated innovation and attention towards the use of telehealth, which often represented the only viable option to provide psychological, behavioural or psycho-educational care services in place of in-person interventions, which were severely reduced or completely ceased at the height of the early phases of the pandemic (Colizzi et al., 2020; White et al., 2021). Telehealth interventions indeed present several benefits, including affordability and potential to reduce barriers to access. Compared with the in-person model and the in-home model, the telehealth model may provide timely and evidence-based support to families who cannot otherwise access traditional services (Ellison et al., 2021; Sutherland et al., 2018) and at the same time reduce costs for both families and the service delivery system (Little et al., 2018). For some users, remotely delivered services may even provide a welcome opportunity to avoid sensory stressors related to travel or waiting time while retaining the benefits of the interventions (Harris et al., 2021). While certain features of interventions directed to young children or adults with more severe communication impairments may be limited in their potential for adaptation to a telehealth format, parent training, psychoeducation, and parent-mediated interventions lend themselves to be adapted for remote delivery with relatively few modifications.

### Evidence of Acceptability, Feasibility and Effectiveness of Telehealth Parent Training

Telehealth has the potential to break several of the existing barriers to service access and has been recommended as a means to connect people to knowledge and training (Lord et al., 2022). However, it may also, by its nature, have additional, novel barriers that may impact its feasibility and acceptability, i.e., respectively, the extent to which the intervention can be carried out as planned across sites and its appropriateness or relevance to users and providers in real-world settings (Smith et al., 2007). Indeed, despite a high need for remotely delivered interventions at the beginning of the COVID-19 pandemic, in Italy, a country hit hard and early on, only under a third of caregivers reported receiving remote support from public healthcare services, schools or private therapists during the first wave of the pandemic, with only a minimal proportion finding such support useful (Colizzi et al., 2020).

A number of studies have examined the acceptability, feasibility and effectiveness of remotely delivered parent training interventions for caregivers of children with ASD. For feasibility, the primary concern when implementing a telehealth service is technological issues. Although prevalence of owning a device (pc, tablet, smartphone) and an internet connection is growing worldwide, the World Bank (2016) has warned against the rising digital disparities between HICs and LMICs, which have been exacerbated even more by the COVID-19 pandemic (Amaral & de Vries, 2020). Across regions and countries with different levels of income there is still a great percentage of disadvantaged families who have no Internet connection (Little et al., 2018; Salomone & Maurizio Arduino, 2017). For telehealth services to be effective, it is recommended that users have a highspeed, wired-cable Internet connection, a laptop with webcam (Lee et al., 2015) and device that can support necessary software. However, this is not always possible as participants may use cellular services that do not ensure a good and stable connection (Gerow et al., 2021) or there may be multiple people in the same home connected to the internet slowing down upload speeds (Pierson et al., 2021). Socioeconomic status is another aspect that can impact the attendance and feasibility of remote intervention, as caregivers may feel overwhelmed by competing responsibilities (Dai et al., 2021). Also, the presence of disruptions and/or other family members who may require attention, interrupt, and interfere with the assessment or intervention (Lerman et al., 2020) may have an impact on feasibility, to the point that families may need to restrict scheduling of appointments to times when siblings are busy or when another parent is present (Gerow et al., 2021). Lower internet skills and general satisfaction with the service providers may also negatively affect participation (Salomone & Maurizio Arduino, 2017).

Conversely, despite such barriers to feasibility, the acceptability to caregivers is usually high (Baharav & Reiser, 2010; Bearss et al., 2018; Dai et al., 2021; Ingersoll & Berger, 2015; Lau et al., 2022; Montiel-Nava et al., 2022; Pickard et al., 2016; Sengupta et al., 2021a; Tsami et al., 2019; Vismara et al., 2018; Wainer et al., 2021) and, generally, caregivers report high levels of satisfaction (Boisvert & Hall, 2014; Pi et al., 2021; Sutherland et al., 2018). Levels of caregiver involvement are satisfactory and greater in therapist-assisted than in self-directed online programs (Pickard et al., 2016). This is relevant as high levels of acceptability and caregiver involvement are associated with greater likelihood of completing the intervention and higher fidelity of strategies implementation (Ingersoll & Berger, 2015; Little et al., 2018). High levels of parent fidelity to intervention and adherence to home practice can be reached (Bearss et al., 2018; Sengupta et al., 2021a).

Finally, some evidence is available regarding the effectiveness of remotely delivered parent training interventions, i.e., their ability to have an effect in a defined and real clinical setting. Systematic reviews show that telehealth services for children with ASD, including parent-training and parent-mediated intervention, are possibly equivalent and comparable to in person services (Boisvert & Hall, 2014; Ellison et al., 2021; Sutherland et al., 2018) or show better outcome than control groups (Ellison et al., 2021); although a metanalysis did not find consistent improvements in the social communication domains (Pi et al., 2021).

Given the mixed picture of advantages and challenges of telehealth, the field would benefit from evidence obtained from comparisons between in-person and online parent training for parents with children with ASD; however, these studies are rare and limited in design. Ashburner et al. (2016) qualitatively explored through interviews with four caregivers and nine service providers their experience of participating in remote and in-person interventions, identifying the following benefits: cost savings, time and travel, flexibility, and convenience; and the following disadvantages: technical issues and difficulties establishing effective communication. Bearss et al. (2018) compared an online delivery of the RUBI parent training for maladaptive behaviour with the pre-specified benchmarks of the original trial (Bearss et al., 2018), reporting comparable feasibility and preliminary efficacy across modalities. Hao et al. (2021) used a non-randomised design to compare an adaptation of Project ImPACT delivered in-person and remotely showing equivalent and comparable outcomes between groups, but acceptability or feasibility were not measured. Lau et al. (2022) examined acceptability and feasibility of the “Caregiver skills training for families of children with developmental delays or disabilities” (CST; Salomone et al., 2019; WHO, 2022) across asynchronous e-learning, fully virtual synchronous and hybrid in-person and virtual delivery modes showing comparable acceptability and satisfaction rates across the three modes, but the study was limited by the reduced sample size and high attrition rates.

### Contributions of the Current Study

There is therefore scant evidence of equivalence or superiority between remotely-delivered caregiver-mediated interventions and in-person delivery modes. This study aims to contribute to address this research gap, adapting an existing intervention to telehealth format and comparing it to existing data from face-to-face delivery. We employed a mixed-method approach to report on the process of adapting and piloting a remote-delivery version of the CST. The CST, which is informed by principles of naturalistic developmental behavioural interventions (NDBI: Schreibman et al., 2015), has shown good acceptability and feasibility in both high- (Lau et al., 2022; Salomone et al., 2021a; Seng et al., 2022) and low-resource contexts (Montiel-Nava et al., 2022; Sengupta et al., 2021b; Tekola et al., 2020). The study aims to examine the implementation of CST delivered remotely in public health services in Italy. Specifically, we aimed to: (1) compare the overall feasibility, acceptability and clinical outcomes data between the ‘virtual’ (i.e., remotely delivered) and in-person delivery modes, with a secondary analysis of data derived from a pilot randomised controlled trial (RCT) of CST delivered in person in public health settings (Salomone et al., 2021b); (2) assess the feasibility and acceptability of novel and adapted components specific to the virtual CST; (3) explore experiences of parents’ and clinicians’ participating in the virtual CST with a qualitative analysis. Such mixed-method approach with multiple informants (caregivers, interventionists and observers) allowed us to utilize the strengths of both the quantitative and qualitative methods, exploiting the qualitative data to validate or refine quantitative findings and quantitative data to explain findings from the qualitative data (Fetters et al., 2013).

## Methods

### Procedure

#### Adaptation of the WHO Caregiver Skill Training for Remote Delivery

Clinical psychologists with experience of CST implementation and a WHO CST Team member adapted the program for virtual delivery. The following factors were considered when devising adaptations that could be feasible and acceptable alternative activities to face-to face activities: (a) rationale and aims of the activities that required adaptation; (b) enhancement of the elements that showed high acceptability and relevance in the in-person delivery mode; (c) technical requirements, barriers and potential advantages of activities delivered via videocall; (d) potential for synchronous interaction between facilitators and parents and among parents to maximise participation. The nine ‘group sessions’ and the three ‘home visits’ were adapted for delivery via group and individual videocalls using a videoconferencing system.

*Group sessions* CST consists of nine group sessions. All core contents remained unchanged. The adaptations concerned the instructional methods and the face-to-face practical activities (Table 1).

**Table 1:** Adaptation of CST Version 3.0 for remote delivery

Standard component	Adaptation
Group sessions	
Brief wellness activity	Unchanged
Home practice review	*Enhanced home practice review with video review* The activity is expanded providing specific feedback with reference to a videorecorded caregiver/child home or play routine shared by the caregiver ahead of the session. To preserve the privacy of participants, videorecordings are not shown but common issues and troubleshooting strategies are highlighted to maximise the benefit to the group. Applies to Sessions 4 and 9 only
Caregiver story (clinical vignette)	Unchanged
Group discussion	Unchanged
Demonstration (live modelling of strategies)	*Videorecorded modelling of strategies* Streaming of pre-recorded videos including an introduction to the strategies followed by a brief scene based on the original scripts
Practice in pairs (caregiver role play of the session’s strategies)	Dropped
Plan for home practice	*Enhanced plan for home practice* To compensate for the lack of hands-on practice the activity is expanded with additional time dedicated to 1:1 discussion of scenarios, possible difficulties and troubleshooting strategies (e.g. “What would you do if your child walked away when you offered him a choice of toys?”). Participants are assigned in two breakout rooms with a facilitator in each
Home visits	
Review of key messages, strategies and home practice and Plan for the guided practice	Unchanged
Guided caregiver/child practice (observation and coaching)	Unchanged
Demonstration of strategies during facilitator/child interaction	Dropped
Review of the guided practice	*Review of the guided practice with video-feedback* The video-recorded caregiver-child interaction is streamed via screen-sharing for the provision of video-feedback. The video is subsequently sent to the caregiver for independent review

*Home visits* CST includes three home visits. The first allows facilitators to get to know the family and set intervention goals; home visits 2 and 3 aim to support the caregivers in implementing the strategies with their child and plan the independent practice. As the COVID-19 containment measures in force at the time of the study only permitted limited in person clinical consultations, the first home visit was carried out in person at the local health care service, rather than at participants’ homes, to facilitate rapport with the family and ensure standardisation of baseline data while minimising sanitary risk. The other two home visits, conducted after Session 5 and after the last group session, were adapted to be delivered as 60 min videocalls via teleconference (Table 1).

### Design and Participants

The pilot implementation of CST delivered remotely (hereafter: ‘virtual CST’) had a pre-test/post-test design; data were collected at baseline and immediately post-intervention. Three editions of the virtual CST were delivered on a first-come, first-served basis. Recommended group size in a CST group is 8 caregivers; in our study, group sizes for the three editions were: n = 8; n = 8; n = 9, for a total sample size of 25 participants. The three groups attended the virtual CST between October 2020 and September 2021, when the restrictions due the COVID-19 pandemic resulted in the closure of schools, working from home arrangements and limitation or cessation of most face-to-face services.

**Fig. 1:**
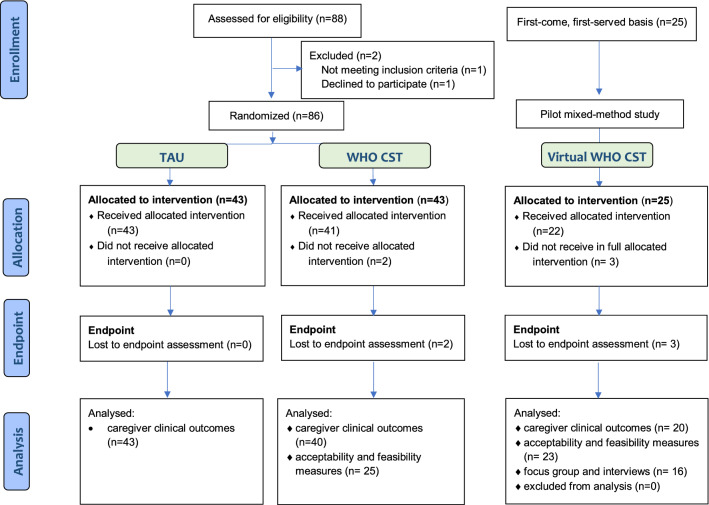
Participant flow

Additionally, we used existing data from a pilot RCT of CST delivered in person (n = 43) against enhanced treatment as usual (TAU; n = 43) previously conducted in the same geographical area in Northern Italy (Salomone et al., 2021a; Salomone et al., 2021b) to compare acceptability, feasibility and preliminary clinical outcomes of the two delivery methods. All participants were caregivers of children with a clinical diagnosis of ASD, recruited through Child Neuropsychiatry units of the National Health Service in the Piedmont region of Italy (Table 2). Both in the pilot of virtual CST and in the original trial, caregivers and facilitators completed feasibility and acceptability measures after each session, and at endpoint took part in focus groups or interviews. Observers completed fidelity ratings. For additional details of the procedure, measures used and description of the intervention procedure in the in-person implementation of CST, see Salomone et al. (2021a). See participants’ flow in Fig. 1.

**Table 2:** Participants characteristics

	TAU (n = 43)		In-person CST (n = 43)		Virtual CST (n = 25)		*p*
	N (%)	M (SD)	N (%)	M (SD)	N (%)	M (SD)	
Child							
Male	34 (79.1%)		33 (76.7%)		23 (92%)		.275
Chronological age (months)		44.21 (9.01)		45.56 (10.06)		55.76 (10.81)	**< .001**
Age at diagnosis (months)		30.43 (6.88)		31.48 (8.63)		35.75 (12.67)	.070
Vineland II composite standard score		55.98 (16.89)		56.98 (15.79)		59.96 (20.21)	.652
Minimally verbal^†^	33 (78.6%)		33 (78.6%)		12 (50%)		**.022**
Primary caregiver							
Female	37 (88.1%)		30 (69.7%)		22 (88%)		.059
Low educational level^‡^	11 (26.2%)		9 (21.4%)		5 (20.8%)		.835
Non-Italian nationality	12 (27.9%)		14 (32.6%)		17 (68%)		**.003**
Capable in basic internet skills^§^	//	//	//	//	21 (87.5%)	4.47 (.79)	
Previous experience of telehealth	//		//		19 (79.2%)		
Low perceived value of telehealth^**¶**^	//		//		15 (62.5%)		

*p* values < 0.05 are marked in bold

^†^Non-verbal or single words. ^‡^elementary or middle school

^§^Score > 3 ‘partly capable’

^¶^Score < 3 ‘the same value’

### Intervention

For virtual CST, teleconferencing platforms (Zoom and Webex) were used to deliver the nine weekly group sessions and the second and third home visits. The first home visit was delivered in person, as explained above. The online intervention was delivered as per the adapted manual by three clinical psychologists and one therapist with experience of CST and previous clinical work with families with children with ASD. Three of the four interventionists (‘facilitators’) had previous experience with the use of videocalls in clinical work and had previously delivered CST in-person having reached minimum fidelity criteria. Participation in the intervention was open to two caregivers per family; however, data were collected on one designated target caregiver. Caregivers used personal devices (smartphone, tablet, laptop, desktop computer) to connect to the sessions: 10 (41.7%) used a smartphone for at least 50% of the group sessions, 14 (58.3%) a laptop or tablet.

The in-person CST was delivered per manual (Salomone et al., 2021b).

### Measures

#### General Baseline Measures

These measures were collected for all participants (in-person CST, treatment as usual, virtual CST).

##### Demographic Questionnaire

Demographic information, including child’s age and age at diagnosis, caregiver age and educational level was collected for children and designated target caregivers.

##### Child Adaptive Behaviour

Parents were interviewed with the Italian version (Balboni et al., 2016) of the Vineland II (VABS, Sparrow et al., 2005), a semi-structured interview that rates the child’s current level of functioning across the domains of Communication, Daily Living and Socialization. Age-normed Standard Scores (mean 100; SD 15) for the Adaptive Behavior Composite (ABC) were used in analyses.

##### Child Language

Caregivers were asked to describe the child’s verbal ability by selecting one of five options (does not talk; uses single words; uses two- or three-word phrases; uses sentences with four or more words; uses complex sentences). Exemplars of each category were given to help respondents.

#### Baseline Measures Specific for Virtual CST

These measures were only collected for caregivers in the virtual CST group.

##### Internet Skills

To measure confidence in the use of the technology we used a rating scale of the Italian National Institute of Statistics (ISTAT) survey on use of new technologies, previously used in Italian samples (Salomone & Arduino, 2016). The items, describing skills such as the ability to read and send emails, visit websites, use a search engine, change browser security settings, do a call or videocall on internet, are rated on a scale from 1 (‘not at all capable’) to 5 (‘fully capable’). Internal reliability was good (Cronbach’s alpha = 0.87).

##### Experience with Telehealth

Caregivers were also asked if they had any previous experience of videocalls with healthcare professionals on a 4-point scale (1 ‘nothing’, 2 ‘one time’, 3 ‘sometime (2–4 times)’, 4 ‘many times (5 or more times)’.

#### General Acceptability and Feasibility Measures

These measures were collected for participants in the in-person CST and virtual CST groups.

##### Attendance

Attendance for each designated target caregiver was registered after each group session and home visit.

##### Caregiver Adherence to Home Practice

To monitor caregivers’ adherence to home practice between sessions caregivers completed an adapted version of *WHO CST Caregiver Diary* rating the frequency, duration, and quality of their practice with CST strategies during the week, on a 5-point scale (1 “not at all true” to 5 “completely true”) and about possible contextual barriers and enactment difficulties. The original questions about frequency, duration of the home practice and frequency of contextual barriers and enactment difficulties remained unchanged; two questions were added about the usefulness of Enhanced plan for home practice, and whether the caregiver had to adjust the plan during the home practice. The questionnaire was completed on the day or the evening before each new group session. The Cronbach’s alpha was excellent (α = 0.95).

##### Interventionist Fidelity of Delivery

Independent observers took part in all group sessions and completed immediately after each session an integrity checklist to evaluate fidelity of delivery of the session’s components (brief wellness activity; home practice review; group discussion; presentation of new content; videorecorded modelling of strategies; enhanced plan for home practice) on a 5-point scale (1 “not delivered to 5 “fully delivered”). The Enhancing Assessment of Common Therapeutic factors (ENACT) scale (Kohrt et al., 2015) adapted for the online delivery, was used to evaluate therapist competence, where for each item on the ENACT a score from 1 ‘need for improvement’ to 4 ‘done well’ is assigned. In the virtual CST, the Cronbach’s alpha was excellent both for the integrity (α = 0.95) and for ENACT (α = 0.96).

##### Feasibility and Acceptability of Standard Intervention Components

Interventionists and parents independently completed after each group session adapted versions of questionnaire included in the WHO CST Monitoring & Evaluation Framework (WHO CST Team, unpublished), rating feasibility and acceptability of the standard intervention components on 5-point scales. Interventionists rated the feasibility for complexity and amount of contents, and for perceived preparedness to deliver the sessions (*WHO CST Post-session Feedback Form for Interventionists*). The acceptability was examined with interventionists’ ratings of alignment with caregiver values, caregiver participation, interest and enthusiasm, comfort and confidence level (*WHO Caregiver Involvement Questionnaire*) and with caregiver self-report ratings of comprehensibility, applicability and alignment with own values (*WHO CST Post-session Feedback Form for Caregiver*s).

For the WHO CST *Post-session Feedback Form for Caregiver*s the Cronbach’s alpha was acceptable (α = 0.77). For interventionists, Cronbach’s alpha was 0.41 for the *WHO CST Post-session Feedback Forms for Interventionists* and excellent (α = 0.97) for the *WHO Caregiver Involvement Questionnaire.*

##### Therapeutic Alliance

The Italian version of the Working Alliance Inventory (WAI; Horvath & Greenberg, 1989) was used to measure caregiver-rated therapeutic alliance at endpoint. The WAI includes 12 items measuring the bond, the tasks and the objectives within the therapeutic relationship on a 7-point scale. Internal reliability was good (Cronbach’s alpha = 0.83).

#### Acceptability and Feasibility Measures Specific for Virtual CST

These measures were only collected for caregivers in the virtual CST.

##### Perceived Value of Telehealth Session

To obtain a measure of perceived value of telehealth, at baseline caregivers were asked to rate the value of a clinical consultation delivered via videocall when no direct assessments are required relative to an equivalent in-person consultation on a 5-point scale (1 ‘much less value’ to 5 ‘much more value’). Subsequently, participants were asked to rate each session (group sessions and home visits) on the same scale.

##### Feasibility and Acceptability of Virtual Group Sessions

For the feasibility of the virtual group sessions interventionists were asked to rate on a 5-points scale the prevalence of factors such as: distracting background noises, issues with the overall audio-visual quality of the call, difficulties with the streaming of the videorecorded demonstrations, difficulties with the assignment of participants in breakout rooms during the enhanced plan for home practice (adaptation of *WHO CST Post-session Feedback Forms for Interventionists).* Similarly, caregivers rated the prevalence of the following factors: distractions (noises in the room or interruptions from family members), technological difficulties which concerned difficulties with the device or the connection, and audio/visual quality of streamed videorecorded modelling of strategies (adaptation of *WHO CST Post-session Feedback Form for Caregivers*).

For acceptability, interventionists rated the comprehensibility, caregiver involvement and relevance of the videorecorded modelling of strategies, the enhanced plan for home practice and the enhanced home practice review with video review (adaptation of *WHO CST Post-session Feedback Form for Interventionists*). Caregivers completed the adaptation of *WHO CST Post-session Feedback Forms for Caregivers* and rated comprehensibility, applicability and realism of the scenes for the videorecorded modelling of strategies. For the enhanced home practice review with video review, caregivers rated the usefulness of discussing the videos and the applicability of strategies suggested for their own child and for the other children. For the enhanced plan for home practice, caregivers rated the comprehensibility and applicability of the activity.

For feasibility data, frequencies of “difficulty reported” (≥ 3), and for acceptability data, frequencies of rating in the “unsatisfactory” (≤ 3) and ‘excessive’ (≥ 4) range are reported.

In this sample, Cronbach’s alpha for adaptation of *WHO CST Post-session Feedback Forms for Caregivers* was excellent (α = 0.93); for interventionists, Cronbach’s alpha was 0.45 for the adaptation of *WHO CST Post-session Feedback Forms for Interventionists*.

##### Feasibility and Acceptability of Virtual Home Visits

Caregivers and facilitators completed a measure of feasibility and acceptability immediately after the end of the videocalls. Facilitators rated the overall feasibility and acceptability of the virtual home visits on 5-point scales (*WHO CST Home Visits Questionnaire for Interventionists*). Additionally, for feasibility they rated the prevalence of the following factors: device or interventionist’s voice distracting for the child or detrimental to engagement during the guided practice, difficulty to clearly see the materials and toys during interaction or to find the right time to intervene during the guided practice, audio-visual quality of the video-feedback and child distracting the caregiver during the video-feedback. For acceptability, interventionists rated the perceived usefulness of the strategies to caregivers and their perceived ease of implementation.

Caregiver rated the feasibility and acceptability of the virtual home visits on 5-point scales (*WHO CST Home Visits Questionnaire for Caregivers*). They rated the prevalence of technological difficulties during the guided practice and the video-feedback, such as difficulties seeing and hearing the video.

For acceptability of the guided practice, to assess whether the device was causing discomfort or interfering with the activity, caregivers were asked about the usefulness of strategies in interaction with the child and the representativeness of their own and the child’s behaviour. For the review of the guided practice with video-feedback, usefulness of reviewing oneself and the child was assessed. Specifically, caregivers were asked if, watching the videorecording, they became aware of own behaviours not noticed before, if they understood better what to change in their behaviour and when to use the strategies with the child, and if they understood better their child’s way to communicate, their behaviour and their focus of attention.

For feasibility data, frequencies of “difficulty reported” (≥ 3), and for acceptability data, frequencies of rating in the “unsatisfactory” (≤ 3) and ‘excessive’ (≥ 4) range are reported.

In our sample Cronbach’s alpha was 0.49 for the WHO CST *Home Visits Questionnaire* for interventionists, and 0.76 for the *WHO CST Home Visits Questionnaire for Caregivers*.

#### Pre–Post Clinical Measures

These measures were collected for all participants (in-person CST, treatment as usual, virtual CST).

##### Parental Stress

The Autism Parent Stress Index (APSI, Silva & Schalock, 2012) was used to measure parental stress. The APSI is a 13 item self-report questionnaire examining parenting stress related to a child’s ASD core deficits, behavioural symptoms, and co-morbid physical symptoms. Internal reliability was good (Cronbach’s alpha = 0.85).

##### Caregiver Knowledge and Skills Test

Parenting self-efficacy and competence were assessed with the *Caregiver Knowledge and Skills Test* (WHO CST Team, unpublished). The tool includes the Caregiver Self-efficacy Questionnaire (CSQ) and the Knowledge and Skills Questionnaire (KSQ). The CSQ is a 13-item 5-point scale measure of parenting self-efficacy applied to domains relevant for parenting a child with developmental delay (e.g., skills development, inclusion, management of challenging behaviour). The KSQ consists of 24 items rated on a 5-point scale and three case-based questions respectively measuring the caregiver’s competence intended as knowledge of the intervention strategies and ability to apply them in a naturalistic context. The case-based questions include a brief clinical vignette of a child and caregiver followed by an open-ended question (e.g. ‘What could this dad do here?’) to probe for intervention strategies (up to six strategies per scenario). The answers are coded and scored for appropriateness by two raters familiar with the intervention strategies (possible score: − 6 to + 6). Counterproductive or detrimental behaviours were scored ‘− 1’, ineffective or unrelated behaviours were scored 0, appropriate and likely effective behaviours were scored as ‘+ 1’; consensus scores were used in the analyses. The Cronbach’s alpha in this sample for CSQ was 0.84 and for KSQ was 0.64.

### Qualitative Assessment of Feasibility and Acceptability of the Virtual Delivery

Immediately after the end of the online delivery, focus groups and telephone interviews were held with all available participants (n = 16) and with all the interventionists (n = 4).

## Data Analysis

A mixed-method was chosen within a convergent and parallel design. Quantitative and qualitative data from multiple informants (caregivers, interventionists, and observers) were individually analysed with both statistical and qualitative analysis. Quantitative and qualitative data were then triangulated through merging in order to validate findings and a contiguous approach was used to present them (Fetters et al., 2013).

Quantitative data were analysed with IBM SPSS Statistics, Version 22. As preliminary analyses, independent sample t tests and chi squares were conducted to verify that the groups did not differ on background variables at baseline. Descriptive analyses were conducted on all acceptability and feasibility measures. Mean values, standard deviations and frequencies below predetermined values corresponding to satisfactory ratings were reported. We compared virtual and in-person CST on all feasibility and acceptability measures in common with t-tests and chi squares. For all variables, the average scores across group sessions and home visits were used. Finally, we compared virtual CST, in-person CST and treatment as usual on preliminary clinical outcomes using between-subjects ANCOVA, adjusting for baseline measures of the outcomes, to evaluate the effects of group membership on immediately post-intervention change scores.

For qualitative data, following the transcription of all audio files, a thematic analysis (Braun & Clarke, 2006) was conducted to identify and analyse recurring themes within the corpus. Initial codes were identified to capture all the aspects that emerged. Subsequently, the codes were organized into broader themes which were given a label that best represented the codes collated.

## Results

The results are reported as per the aims of this study: (1) to compare the overall feasibility, acceptability and clinical outcomes data between the ‘virtual’ and in-person delivery modes; (2) to assess the feasibility and acceptability of novel and adapted components specific to the virtual CST; (3) to explore experiences of parents’ and clinicians’ participating in the virtual CST with a qualitative analysis.

### Baseline Characteristics

In the virtual CST group, basic internet skills were satisfactory (M = 4.47, SD = .79). 19 (79.2%) participants had previous experience with videocalls with healthcare professionals. Regarding background characteristics, while the TAU and the in-person CST groups did not differ on any of the baseline measures, the virtual CST group differed on child age and caregivers’ nationality (Table 2). Baseline levels of outcome measures are reported in Table 5.

### Comparison on Overall Acceptability, Feasibility, and Clinical Outcomes

We compared in-person and virtual adaptation of CST on feasibility and acceptability (respectively Tables 3, 4).

**Table 3:** Feasibility of standard intervention components: comparison between in-person and virtual CST

	In-person CST		Virtual CST		*p*
	N (%)	M (SD)	N (%)	M (SD)	
Overall feasibility					
Integrity		87.98% (5.74%)		90.33% (4.04%)	.396
N. of drop-outs	2 (4.65%)		3 (12%)		.262
High attendance^†^	33 (84%)		20 (90%)		.484
High adherence to home practice^‡^	21 (53.84%)		13 (59%)		.692
Caregiver ratings					
Contextual barriers to home practice^§^					
Unexpected circumstances	21 (52.5%)		5 (20%)		**.007**
Interruptions	11 (27.5%)		3 (12%)		.139
Remembering to practice	5 (12.5%)		3 (12%)		.952
Lack of time	24 (60%)		8 (32%)		**.021**
Enactment difficulties in home practice^§^					
Did not know what to do	2 (5%)		1 (4%)		.851
Did not understand the strategies	3 (7.5%)		1 (4%)		.567
Did not feel confident	11 (27.5%)		3 (12%)		.139
Strategies not appropriate	4 (10%)		3 (12%)		.800
Difficulties engaging child	19 (47.5%)		9 (36%)		.362
Facilitator ratings					
Complexity of contents^¶^		3.09 (.56)		3.28 (.56)	**.048**
Amount of contents^¶^		3.44 (.85)		3.52 (.74)	.574
Preparedness to deliver^⁂^		3.34 (.60)		3.25 (.65)	.381

*p* values < 0.05 are marked in bold

^†^Attendance ≥ 75% of the program

^‡^At least 3–4 times per interval on 75% of intervals

^§^Score ≥ 3 in at least 4 sessions

^¶^Value = 3 ‘at the right level’

^⁂^Values = 3 ‘appropriate’

**Table 4:** Acceptability of standard intervention components: comparison between in-person and virtual CST

	In-person CST		Virtual CST		*p*
	N (%)	M (SD)	N (%)	M (SD)	
Overall acceptability					
Therapeutic alliance		6.22 (.62)		6.26 (.70)	.851
Caregiver ratings^‡^					
Comprehensibility	4 (1.9%)	4.80 (.44)	18 (10.2%)	4.56 (.70)	** < .001**
Applicability	18 (8.4%)	4.54 (.72)	29 (16.4%)	4.32 (.81)	**.004**
Alignment with the values	0 (0%)	4.80 (.39)	13 (7.3%)	4.66 (.62)	**.007**
Facilitator ratings^‡^					
Caregiver participation	2 (2%)	4.59 (.53)	3 (5.6%)	4.35 (.58)	**.012**
Caregiver agreement	4 (3.9%)	4.64 (.55)	2 (3.7%)	4.80 (.49)	.080
Caregiver comfort level	1 (2.3%)	4.40 (.45)	0 (0%)	4.22 (.48)	.150
Caregiver confidence level	2 (4.7%)	4.04 (.62)	0 (0%)	4.09 (.47)	.715
Caregiver enthusiasm	1 (2.3%)	4.39 (.49)	0 (0%)	4.31 (.45)	.544

*p* values < 0.05 are marked in bold

^‡^Values ≤ 3

Mean integrity ratings of the group sessions in the in-person delivery (range 82–97%) did not significantly differ from those in the online delivery (range 86–94%) (*p* = .55). ENACT competency ratings of virtual CST ranged from 82 to 96% across sites (ICC 94.6%); data were not available for in-person CST.

Attendance and drop-out rates were not significantly different across the two delivery methods. In the online delivery group three caregivers dropped out due to religious commitments, conflicts with work schedule and issues with childcare during lockdown, respectively after the first home visit, after Session 2 and after Session 5. In the in-person delivery group, two caregivers dropped out after the first home visit for substance abuse, chaotic life patterns and high levels of parental conflict, as reported by them. The two groups did not differ for intensity of adherence to the home practice nor for reported levels of enactment difficulties in the practice, but caregivers in the in-person group endorsed significantly more frequently unexpected circumstances and lack of time as contextual barriers to the practice. Contents were rated by facilitators as significantly more complex to deliver in the online delivery but there was no difference in the evaluation of the amount of content (Table 3).

Regarding acceptability, caregivers in the online group rated the contents significantly less comprehensible, less applicable and less aligned with values, compared to caregivers who received in-person delivery. In the virtual CST, facilitator ratings of caregiver participation were significantly lower compared to those in the in-person group. No other significant differences emerged. The two groups did not differ in therapeutic alliance, comfort and confidence levels (Table 4).

Table 5 shows the results of the ANCOVA analysis examining the effect of group membership (virtual CST, in-person CST, treatment as usual) on pre-post intervention change scores of clinical outcomes, after controlling for baseline scores. There was a large and significant effect of group membership on caregiver competence, measured with the *KSQ* (η^2^ = .131)*.* Post hoc analyses performed with a Bonferroni adjustment showed that the in-person and the virtual CST groups had both significantly improved more than the TAU group but did not differ from each other. No statistically significant effects emerged for parental stress, measured with the APSI nor for parental self-efficacy, measured with the CSQ.

**Table 5:** Preliminary clinical outcomes: comparison between in-person and virtual CST

	TAU (n = 43)			In-person CST (n = 43)			Virtual CST (n = 25)			F (df)	η^2^	p
	Baseline	Endpoint	Change	Baseline	Endpoint	Change	Baseline	Endpoint	Change			
	M (SD)	M (SD)	M (SD)	M (SD)	M (SD)	M (SD)	M (SD)	M (SD)	M (SD)			
APSI	30.51 (8.80)	30.57 (9.47)	− 0.46 (7.23)	29.07 (8.93)	26.57 (7.38)	− 2.36 (6.47)	28.91 (7.41)	29.10 (9.13)	0.50 (5.01)	2.07 (2)	0.042	.132
KSQ	97.31 (8.36)	97.71 (6.96)	.585 (7.30)^a^	99.27 (7.40)	104.47(9.58)	4.87 (7.72)^b^	96.87 (6.87)	102.95 (8.08)	6.05 (7.26)^b^	6.76	.131	**.002**
CSQ	47.79 (7.14)	48.30 (8.10)	.511 (7.31)	47.10 (8.03)	50.82 (7.16)	3.67 (7.46)	49.67 (7.14)	53.05 (8.02)	2.70 (6.65)	2.706	0.052	.072

*p* values < 0.05 are marked in bold

Means from baseline and endpoint, mean change difference, effect size and *p*-values are from the ANCOVA controlling for outcome measured at baseline. A *p*-value < 0.05 was considered statistically significant. Means in a row without a common superscript letter are statistically different, otherwise are not statistically different (*p* < 0.05)

*TAU* treatment as usual; *CST* Caregiver Skills Training; *ANCOVA* analysis of covariance; *APSI* Autism Parent Stress Index; *CSQ* Caregiver Self-efficacy Questionnaire; *KSQ* Knowledge and Skills Questionnaire

### Feasibility and Acceptability of Novel and Adapted Intervention Components in Virtual CST

#### Perceived Value of Telehealth Sessions

At baseline, 15 (62.5%) caregivers attributed a lower value to videocalls compared to in-person clinical contacts (score < 3; *M* = 2.38, *SD* = .49). Considering the averaged ratings across the nine virtual group sessions and two virtual home visits, 14 (58.3%) caregivers rated online group sessions and 13 (59.1%) caregivers rated home visits via videocalls of less value than an in-person interaction (score < 3; *M* = 2.60, *SD* = .76; *M* = 2.50, *SD* = .74).

We then examined through paired-samples t-tests, the difference between baseline values and average values of group sessions and home visits measured as average, respectively, of the ratings attributed to the nine group sessions and two home visits delivered remotely. There were no statistically significant differences (respectively: *t*(22) = − 1.48 (22), *p* = .076; *t*(22) = − .476 (21), *p* = .32).

### Feasibility and Acceptability of Virtual Group Sessions

Facilitator-rated feasibility and acceptability of group sessions were above satisfactory levels (*M* = 4.33, *SD* = .516; *M* = 5.00, *SD* = .00), although issues with technology were reported. 50% of facilitators reported difficulties with screen sharing and video streaming, and 13.7% of caregiver reported technological difficulties. Distractions and interruptions from other family members were reported in 23.4% of ratings across the nine group sessions. Most caregiver and facilitator acceptability ratings for adapted components were in the ‘good’ range, however ‘unsatisfactory’ for perceived realism of the videorecorded modelling of strategies by caregivers (*M* = 3.32, *SD* = 1.18) (Table 6).

**Table 6:** Feasibility and acceptability of virtual group sessions

	‘Difficulty reported’^†^/‘Unsatisfactory’^‡^	
	N (%)	M (SD)
Feasibility		
General		
Facilitator-rated overall feasibility of delivery	0 (0%)	4.33 (.516)
Facilitator-rated prevalence of distractions	2 (33.3%)	1.83 (1.32)
Facilitator-rated prevalence of technological difficulties	1 (16.7%)	2.04 (.82)
Caregiver-rated prevalence of distractions and interruptions from family members	41 (23.4%)	1.95 (1.30)
Caregiver-rated prevalence of technological difficulties	24 (13.7%)	1.53 (1.00)
Videorecorded modelling of strategies		
Facilitator-rated difficulties with screen sharing	3 (50%)	2.33 (.81)
Caregiver-rated audio/visual quality	37 (23.7%)	1.82 (1.30)
Enhanced home practice review with video review		
Caregiver did not share home video	3 (14%)	
Caregiver shared one home video	6 (27%)	
Caregiver shared two home videos	13 (59%)	
Caregiver-rated prevalence of interruptions	4 (8.5%)	
Caregiver-rated prevalence of difficulty to record video	4 (8.5%)	
Caregiver-rated prevalence of difficulty to share video	5 (10.6%)	
Caregiver-rated prevalence of lack of time to practice	5 (10.6%)	
Enhanced plan for home practice		
Facilitator-reported difficulty in assignment of participants in breakout rooms	2 (33.3%)	1.83 (1.32)
Caregiver-reported adjustment of home practice plan	61 (34.9%)	2.14 (1.32)
Acceptability		
General		
Facilitator-rated overall acceptability	0 (0%)	5.00 (.00)
Videorecorded modelling of strategies		
Facilitator-rated comprehensibility	14 (29.2%)	3.33 (.63)
Facilitator-rated involvement	3 (6.3%)	4.29 (.58)
Facilitator-rated relevance	12 (25%)	4.13 (.78)
Caregiver-rated comprehensibility	32 (14%)	4.41 (.88)
Caregiver-rated applicability	40 (25.5%%)	4.19 (.94)
Caregiver-rated realism	92 (58.6%)	3.32 (1.18)
Enhanced home practice review with video review		
Facilitator-rated comprehensibility	0 (0%)	3.00 (.00)
Facilitator-rated involvement	0 (0%)	4.67 (.49)
Facilitator-rated relevance	2 (16.7%)	4.58 (.79)
Caregiver-rated usefulness	4 (11.1%)	4.45 (.84)
Caregiver-rated applicability of strategies	5 (13.9%)	4.29 (.90)
Enhanced plan for home practice		
Facilitator-rated comprehensibility	15 (30%)	3.30 (.46)
Facilitator-rated involvement	0 (0%)	4.76 (.43)
Facilitator-rated relevance	2 (4%)	4.58 (.57)
Caregiver-rated comprehensibility	18 (10.3%)	4.55 (.73)
Caregiver-rated applicability	33 (18.9%)	4.28 (.84)

^†^Value ≥ 3 for all items except for overall feasibility (score < 3 ‘partly feasible’)

^‡^Values ≤ 3 for all items except for facilitator-rated comprehensibility (values ≥ 4 ‘Excessive’)

### Feasibility and Acceptability of Virtual Home Visits

Facilitator-rated feasibility of home visits were in the ‘satisfactory’ range (*M* = 3.83, *SD* = .40), but the facilitators were able to deliver video-feedback to only 72.1% of caregivers due to technical difficulties. Facilitator-rated acceptability of home visits were above satisfactory levels (*M* = 4.50, *SD* = .54).

Most caregiver-acceptability ratings for home visits delivered online were in the ‘good’ range, but in the ‘unsatisfactory’ range for representativeness of child behavior during the home visits (*M* = 3.76, *SD* = 1.12). (Table 7).

**Table 7:** Feasibility and acceptability of virtual home visits

	‘Difficulty reported’^†^/‘Unsatisfactory’^‡^		
	N (%)		M (SD)
Feasibility			
General			
Facilitator-rated overall feasibility	1 (16.7%)		3.83 (.40)
Guided practice during home visits			
Facilitator-report: child distracted by camera/voice	6 (14%)		1.58 (1.02)
Facilitator-report: play materials or actions off screen	2 (4.7%)		1.53 (.73)
Facilitator-report: difficulty knowing when to intervene	6 (14%)		1.63 (.92)
Review of guided practice with video-feedback			
N of video-feedback	31 (72.1%)		
Facilitator-rated quality of video-feedback	1 (3.22%)		2.08 (.36)
Facilitator-report: child distracts the caregiver	11 (25.6%)		1.95 (1.19)
Caregiver-report: technological difficulties	1 (3.3%)		1.15 (.73)
Acceptability			
General			
Facilitator-rated overall acceptability	0 (0%)		4.50 (.54)
Guided practice during home visits			
Facilitator-rated usefulness of strategies for caregiver	6 (14%)		4.09 (.81)
Facilitator-rated quality of strategies implementation	10 (23.3%)		3.83 (.85)
Caregiver-rated usefulness of strategies in interaction with child	1 (2.4%)		4.69 (.49)
Caregiver-rated representativeness of child behaviour	17 (40.5%)		3.76 (1.12)
Caregiver-rated: representativeness of caregiver behaviour	8 (19%)		4.31 (.89)
Review of guided practice with video-feedback			
Facilitator-rated usefulness of videofeedback for caregiver	3 (10%)		4.06 (.65)
Caregiver-rated usefulness of reviewing own behaviour	4 (13.8%)		4.16 (.78)
Caregiver-rated usefulness of reviewing child’s behaviour	2 (6.9%)		4.31 (.76)

^†^Value ≥ 3 for all items except for quality of video-feedback (3-point scale, value = 1 terrible), overall feasibility (score < 3 ‘partly feasible’)

^‡^Values ≤ 3 for all items

### Qualitative Analysis of Parents’ and Clinicians’ Experiences of the Virtual CST

**Fig. 2:**
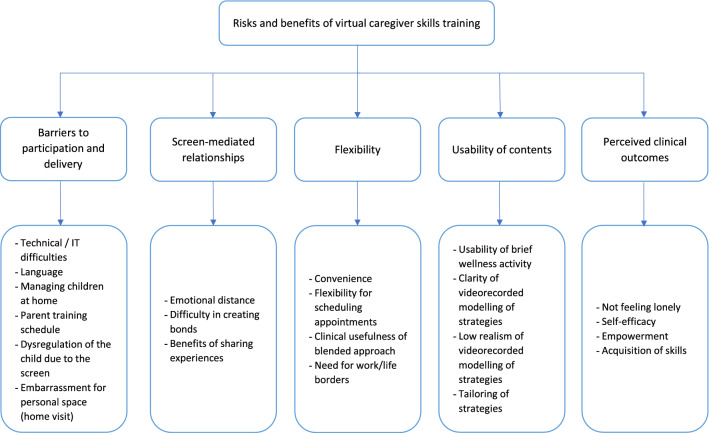
Thematic analysis: risks and benefits of virtual caregiver skills training

Five main themes emerged from the thematic analysis, as detailed below (Fig. 2 and Supplementary Materials).

#### Barriers to Participation and Delivery

Several aspects emerged that made it difficult for the parents to fully participate and for the facilitators to deliver the parent training’ contents, mostly concerning the lack of technological expertise (for facilitators, being able to allocate participants to breakout rooms or to share a videorecorded file during video-feedback) and difficulties experienced with the internet connection. The resulting poor audio-visual quality particularly affected non-native Italian participants, as it exacerbated their existing difficulties in comprehension of the Italian language. Furthermore, participation from home, rather than a neutral space, made it more difficult for parents to focus on the group sessions when children were present (especially during periods of strict lockdown), while at the same time it did not fully eliminate conflicts with work commitments. During the virtual home visits, both parents and facilitators reported that children often became dysregulated because they were either distracted by the voice of the facilitator or excessively attracted to the devices used in the videocall. Some parents voiced feeling embarrassment for exposing the ‘mess’ in the house or self-consciousness during the caregiver/child interaction.

#### Screen-Mediated Relationships

The quality of the bonds built, and the emotions felt within the interactions among parents and between parents and facilitators were described ambivalently. Several caregivers reported having felt ‘distant’ from the others and the difficulty in creating bonds with the other participants. At the same time they recognised that, despite its limitations, the online group represented an enriching possibility of sharing experiences, difficulties and tips with other parents. When faced with the emergence of important and emotionally charged issues for caregivers, a sense of helplessness and perceived emotional distance was also reported by facilitators.

#### Flexibility

The virtual CST was described as more flexible than traditional in-person services. This included several strengths related to the elimination of travel time: for caregivers, this meant the possibility to fit more easily the parent training around other personal or work commitments, and for facilitators the more efficient scheduling of multiple individual home visits appointments in the same day, and the lesser impact of re-scheduling in case of unforeseen events. The hybrid formula combining an in-person first home visit at the clinical centre with remote delivery of the other sessions was reported as a positive and essential delivery mode by facilitators to have an example of the child’s functioning. However, the increased flexibility meant the line between personal life and external commitments was perceived as more blurred. Facilitators lamented that parents tended to consider them as ‘always reachable’ online, even during non-working hours. Some caregivers reported that the elimination of travel time, while economically more convenient, ultimately resulted in a loss of ‘extra’ time that could have been dedicated to brief wellness or leisure activities for self or the couple (such as unwinding during a car trip or dining out after the group session).

#### Usability of Contents

Caregivers found contents and activities mostly usable. The brief wellness activity (breathing exercise) was considered helpful by about half of the parents while some reported that they did not feel comfortable in participating. The videorecorded modelling of strategies was found to be clear, and the initial explanation of the strategies was helpful for caregivers, but the scenes were considered not realistic enough. Finally, the tailoring of strategies during the group discussions and home visits was unanimously considered a positive element.

#### Perceived Clinical Outcomes

Parents reported several perceived positive effects of taking part in the program, including: a sense of relief in sharing the same challenges with other caregivers; an increased sense of self-efficacy; a sense of empowerment and greater awareness of what could be done to help their children; the acquisition of strategies that they could use during everyday interactions with their children.

## Discussion

In the context of the severe disruptions to service access due to the COVID-19 emergency (White et al., 2021), we developed and piloted an adaptation of CST for virtual delivery using videoconferencing software and video-recorded demonstrations. We compared feasibility, acceptability and preliminary clinical outcomes data of our pilot of virtual CST, delivered during a period of full lockdown in Italy, with existing data derived from a pilot RCT of CST delivered in person. Our aim was to explore whether the online delivery method could represent a longer-term response to the needs of families of children with neurodevelopmental disorders, beyond the current health emergency.

Overall, virtual CST was found to be feasible and accept to caregiver and facilitators, in line with several previous studies that evaluated the acceptability of online parent training interventions for ASD (Baharav & Reiser, 2010; Bearss et al., 2018; Dai et al., 2021; Ingersoll & Berger, 2015; Lau et al., 2022; Montiel-Nava et al., 2022; Pi et al., 2021; Pickard et al., 2016; Sengupta et al., 2021a; Tsami et al., 2019; Vismara et al., 2018; Wainer et al., 2021). The online adaptation of CST was delivered with high levels of observer-rated competency and integrity, not differently from the in-person CST.

There were no differences in caregivers’ rates of drop-out and attendance between the virtual and in-person CST, but exit interviews revealed that in the virtual CST group drop-outs were driven by the restrictions of lockdown, rather than by extreme family characteristics as was the case in the in-person CST. Moreover, the triangulation of qualitative and quantitative data highlights a complex picture with reference to the barriers to attendance. Depending on co-occurring COVID-related school closures, childcare availability, and smart-working arrangements, the virtual delivery was perceived either as a barrier to participation (when the parent felt distracted by the child or unable to ‘switch off’) or conversely an enhancing factor, thanks to the convenience of not having to travel, as previously emerged for rural/remote areas (Owen, 2020; Sengupta et al., 2021a), and to the benefits of taking the course together with other parent. The high attendance in virtual CST may have been supported by the overall satisfactory facilitator- and caregiver-rated dimensions of acceptability and facilitator-rated caregiver involvement, consistent with previous findings (Little et al., 2018).

In virtual CST the therapeutic alliance and caregiver’s comfort and confidence levels were satisfactory and similar to those of in-person CST, unlike previous reports (Owen, 2020). However, although several studies have reported good levels of acceptability related to intervention delivered via telehealth (Pickard et al., 2016; Sengupta et al., 2021a), in virtual CST caregiver participation was lower and contents were overall found to be less acceptable by caregivers, as well as more complex to deliver by facilitators. This may be due to the fact that the virtual CST group disproportionately included parents of non-Italian nationality, who reported indeed that their difficulties with understanding the language were worsened by technological issues, as previously found (Klein et al., 2021; Lee et al., 2015). In our study we report lower ratings of caregiver participation in virtual CST compared to in-person CST, mirrored by qualitative findings of emotional distance and impotence perceived by facilitators and caregiver difficulties in creating meaningful relationships in virtual CST. These findings are in line with those of Montiel-Nava et al. (2022), who reported that parents receiving CST remotely in rural Missouri seemed more focused understanding the content than in sharing experiences with other parents, and of Taylor et al. (2021), who reported greater reluctance to ask questions, participate in group discussions and connect with other participants in parents enrolled in an online parent training than in those receiving the corresponding in-person training. However, it is important to consider that our findings may also have been affected by emotional reactions to isolation during the pandemic. An additional in-person group meeting at the end of the training was suggested by facilitators for future implementations as an opportunity for socialising as well as taking time for themselves and the couple.

Regarding the feasibility of delivery, issues with use of technology and poor audio-visual quality of the group sessions and home visits were some of the barriers reported by most caregivers, particularly in one site by facilitators, as previously found (Bearss et al., 2018; Gerow et al., 2021; Harris et al., 2021; Lee et al., 2015; Lerman et al., 2020). One potential troubleshooting strategy to ensure effective access could be the provision of devices, internet subscriptions or access to a private space with free internet access, as previously suggested (Little et al., 2018); however, these are potentially non-sustainable options for community implementation within public health services.

We then considered feasibility and acceptability of novel or adapted intervention components for online delivery. With respect to these adapted activities during the group sessions, the triangulation of qualitative and quantitative data highlights a low realism of the videorecorded modelling of strategies and a good acceptability of the Review of the Home Practice based on feedback provided on video of the caregiver-child interaction.

The videorecorded modelling of strategies, which substituted the live demonstration, was rated as low in realism, as supported by qualitative data, with the ‘child’ (an adult facilitator) considered too compliant. This is not surprising, since the original scripts, which had been on occasion rated low in acceptability in the in-person implementation (Salomone et al., 2021a), were not modified. Producing realistic and accurate role plays, including the use of realistic props and environments and realistic child behaviours (that is, not ‘too compliant’) is challenging, and can negatively affect caregivers’ perception of the feasibility of implementation of intervention strategies, particularly in self-directed models (Dai et al., 2021). Additionally, our novel adapted component of the review of the home practice based on feedback provided on a video of a caregiver-child interaction proved challenging in feasibility. Despite the high acceptability ratings across dimensions both by facilitators and caregivers, it is notable that, due to difficulties in recording or sharing video files (as previously found in Pierson et al., 2021), and due to the lack of time (the most frequent barrier for caregivers in Klein et al., 2021), a significant portion of caregivers (41%) were not able to share the recording in at least one of the two planned occasions.

Technical issues and cumbersome processes to share the videorecording also affected the video-feedback during the virtual home visits, resulting in lack of delivery in a third of opportunities. When video feedback was feasibly delivered, caregivers found it highly useful to have reviewed themselves and their child, as previously reported (Klein et al., 2021), but reported that the presence of the camera and the interventionist’s voice during the direct coaching were interfering with the child’s spontaneous behaviour, thus affecting its representativeness. Communicating via Bluetooth headphones or the videoconferencing chat to guide the caregiver and placing the device out of the child’s sight were put forward as possible strategies by facilitators, as suggested by others (Lerman et al., 2020).

As in the study by Klein et al. (2021), another significant barrier was the presence of other family members during the group session and home visits and the consequent need to have another adult present to take care of the child(ren), for example during video-feedback at the end of home visits. There is indeed evidence that during the pandemic high levels of co-parenting acted as a buffer for parental stress (Bentenuto et al., 2021); specifically, another adult may provide important practical support and childcare during direct coaching sessions (Gerow et al., 2021; Shire et al., 2021). The pilot of virtual CST was conducted during periods of full lockdown, therefore the feasibility of virtual home visits and group sessions under normal circumstances may be different.

Aside from the challenges discussed above, we identified several advantages of virtual CST which may be applicable to other telehealth caregiver-mediated interventions. First, the combination of both online and in-person elements was positively received by caregivers and facilitators. The format for telehealth programs is usually fully remote, although parents may prefer a blended model (Ashburner et al., 2016; Owen, 2020; Sengupta et al., 2021a). We piloted a hybrid model, with the first home visit delivered in person (albeit at the clinic rather than at participants’ homes) and all other components delivered remotely. Blended interventions, as suggested in the Hall and Bierman’s review (2015), could promote caregivers’ engagement and positive outcomes and combine personalization of the intervention, flexibility, and cost-effectiveness. In our study clinicians praised the advantages of the first in-person contact for rapport building, skills assessment and goal-setting as in Ashburner et al (2016), and reported that the remote home visits allowed facilitators to see the child in a naturalistic environment without being intrusive, as previously found (Baharav & Reiser, 2010; Klein et al., 2021), favouring a more active parent participation (Klein et al., 2021; Meadan & Daczewitz, 2015). Similarly, Lau et al. (2022) showed that a hybrid delivery of CST (with virtual group sessions and in-person home visits) received high satisfaction ratings, comparable to both the synchronous online and the asynchronous e-learning delivery, but was reported to be more acceptable and feasible than the other delivery modes in focus groups with parents and facilitators. Lastly, as in Klein’s study (2021), some caregivers reported embarrassment or self-consciousness, however not differently from reports of in-person home visits (Salomone et al., 2021a).

A second positive element concerns the adherence to home practice. We not only report high levels of caregiver adherence, not different from those of in-person CST, but also fewer parent-reported ‘contextual’ barriers, such as lack of time or the presence of unexpected circumstances, compared to in-person CST. This may be explained by the restrictions to social gatherings in response to the pandemic, which despite their negative effects on parental stress (Bentenuto et al., 2021; Kong, 2021; Manning et al., 2021; White et al., 2021), also reduced commitments, evening activities and travel (Taylor et al., 2021) allowing families to spend more time together. It is also noteworthy that there were no differences in reported ‘enactment’ barriers (difficulties in understanding and confidently apply the intervention strategies) in the home practice between the online- and in-person CST. This may be an indication that the modified plan for home practice in the virtual CST group sessions still allowed the same level of understanding and confidence as in the in-person CST, despite lacking the live role play component, as supported by the high comprehensibility and relevance caregiver ratings of this activity. This is in line with findings of Bearss et al. (2018), who found high rates of engagement and satisfaction despite having dropped home visits and role play.

In our sample, 79% of caregivers had previously used telehealth interventions, and at baseline 62.5% of these reported a perception of a lesser value of telehealth compared to in-person consultations, even when the type of consultation did not necessarily require in-person interaction. This negative perception was maintained through participation in the intervention, as shown in the non-significant pre-post comparisons. It is therefore notable that, despite this and the above-mentioned barriers to participation, caregivers and facilitators identified several advantages of the online delivery in the focus groups. As detailed above, advantages included both practical and organizational factors and emotional factors (flexibility, benefits of sharing experiences, not feeling lonely, good therapeutic alliance), as similarly reported (Fisher et al., 2020; Sengupta et al., 2021a).

Finally, in this study we compared pre–post change on clinical outcomes in virtual CST, in-person CST and TAU. We report a large and significant group effect for caregiver competence, indicating that both the in-person and the virtual CST significantly improved more than the TAU group and were not different from each other. However, there were no statistically significant group effects for parental stress nor for parental self-efficacy when comparing virtual CST, in-person CST and treatment as usual. This suggest that, in spite of the challenges described above, the online delivery of CST may still lead to an increase in knowledge of strategies that caregivers can use in interaction with the child. Our finding is in line with previous evidence of increased parental knowledge and use of intervention strategies in online parent trainings (Dai et al., 2021; Montiel-Nava et al., 2022; Parsons et al., 2017; Wainer & Ingersoll, 2013). However, considering that the online and in-person CST were delivered under very different circumstances, this does not suggest equivalence of the two modalities. For self-efficacy, there was a nominal increase in each group over time, however no between group differences were identified. It is possible that the elimination of the live practice of intervention strategies in role play activities was not buffered by the addition of the enhanced plan for the home practice and the review of home videos, leading parents to feel not fully capable to utilize the strategies, despite the actual increase in caregiver competency. Finally, it is not possible to exclude that participation in the program at a time of increased parental stress (Bentenuto et al., 2021; Kong, 2021; Manning et al., 2021; White et al., 2021) and concerns for newly emerging problem behaviours or loss of skills (Wang, 2021), may have interfered with the effect of the intervention. This consideration applies to all clinical outcomes but have a particular relevance for parental stress.

### Strengths, Limitations and Future Directions

The combination of quantitative and qualitative methods to examine in detail acceptability, feasibility and preliminary indicators of effectiveness of a community-based intervention delivered online and in-person can be considered methodological strengths of this study. However, our findings should be considered in the context of some design limitations. We relied on a secondary analysis of the Salomone et al. (2021b) trial to compare online and in-person delivery modes as it was deemed unethical to randomly assign participants to a ‘treatment as usual’ condition considering that the study was conducted at the height of COVID-19 restrictions, when most services were unavailable or substantially reduced in Italy (Bentenuto et al., 2021). Therefore, the lack of randomization, the striking differences of the historical periods in which the data for the in-person and virtual intervention were collected (during the pandemic and ‘normal time’), differences in some of the baseline characteristics and uneven across groups sample sizes warrant caution in the interpretation of the findings. Additionally, restrictions in place to face-to-face contacts due to the pandemic and ethical considerations to limit the burden of assessments of families prevented the collection of high-quality direct observational data and clinician direct assessments.

Future studies should build on the promising findings with respect to feasibility, acceptability and preliminary outcomes to formally examine the effectiveness of virtual CST against in-person delivery with a randomised controlled design, at a time when there are no restrictions in place to face-to-face contacts and employing blind rated outcomes from direct observational measures and assessments.

## Conclusions

Taken together, this pattern of findings indicates that virtual CST is an overall feasible and sufficiently acceptable, albeit partial, clinical response to the needs of families. This modality could therefore represent an option to allow continuity of care and cost reduction not only during health or other emergencies that prevented delivery of face-to-face services, but potentially also in other contexts where in-person delivery is not possible, due to geographical distance, restrictions to travel or personal circumstances.

### Supplementary Information

Below is the link to the electronic supplementary material.

Supplementary file1 (DOCX 18 kb)
