# Supplementary material for: Supporting Caregivers Remotely During a Pandemic: Comparison of WHO Caregiver Skills Training Delivered Online Versus in Person in Public Health Settings in Italy
Source: J Autism Dev Disord. 2022 Dec 1;54(2):765–84. doi: 10.1007/s10803-022-05800-y (PMC9713075; doi:10.1007/s10803-022-05800-y)
Supplement: Supplementary file 1 — Supplementary file1 (DOCX 18 kb) [file 10803_2022_5800_MOESM1_ESM.docx]

Supporting caregivers remotely during a pandemic: comparison of WHO Caregiver Skills Training delivered online versus in person in public health settings in Italy

*Supplementary Materials*

*Themes and subthemes derived from the qualitative analysis of focus groups and interviews*

| *Barriers to participation and delivery* | |
| --- | --- |
| Technical / IT difficulties | Ca: *‘I had a lot of difficulty to see the Demonstration Video […] I saw it after the meeting’*  Fa: *‘There was a mother who did not usually speak but she had an audio problem, because when she spoke there was the echo, so she preferred to be silent’* |
| Language | Ca*: 'For me who do not speak the language it’s a bit more difficult because I have to pay close attention, sometimes the words cannot be heard clearly'* |
| Managing children at home | Ca: *‘My son was present at home, he often heard me speak and wanted to intervene and enter the room while I was attending the meeting, here is the difficulty, let's say, in managing the child during the meeting’* |
| Parent training schedule | Ca: *‘This schedule, in the morning from 9.30 to 12:00, honestly for those who work, was a backbreaking thing’* |
| Dysregulation of the child due to the screen | Ca: *‘We struggled to make the video calls because my daughter refuses the mobile phone / computer, she never wanted to be filmed [...] we were able to send videos because we hid the phone’* |
| Embarrassment for personal space (home visit) | Ca: *‘I didn’t want to show the mess on camera, that was my concern’*  Ca: *‘There was a bit of tension and embarrassment at the beginning’* |
| *Screen-mediated relationships* | |
| Emotional distance | Ca: *‘I consider screen-mediated relationships as something cold, aseptic […] When I look a person in the eyes, face to face – I don’t know – maybe I establish a more empathic relationship’*  Fa: *‘I feel a sense of helplessness because I am present, but they are so far and distant and therefore I feel I cannot do much’* |
| Difficulty in creating bonds | Ca: *‘I am sure that if I had gone to the [clinical centre] to attend parent training in person, already the first day I would have exchanged my telephone number with the other parents, immediately’* |
| Benefits of sharing experiences | Ca: *‘The discussion with other parents is certainly very useful, both to understand the various facets of this problem, and also to exchange some suggestions, this is very useful’* |
| *Flexibility* | |
| Clinical usefulness of blended approach | Fa: *‘It is essential to have an initial meeting only with the family, preferably in person, because you really need to have examples of the child’s functioning’* |
| Convenience | Ca: *‘It helped me a lot because I have very strange working hours and so being able to unplug from a business call and immediately hook up to the parent training was wonderful, I optimized the times a lot and in some cases it allowed me not to skip sessions'* |
| Flexibility for scheduling appointments | Fa: *‘It happened that a video call lasted just 5 minutes. The child was not in fine form, so we stopped the video, chatted with her mom for a while and then said goodbye. If we had gone there in person, Mom would have been much more frustrated about what happened’* |
| Need for work/life borders | Fa: *‘Being available online often means that parents send messages or call even on Saturday nights to keep up with all the pieces (sending videos, compilation of questionnaires, changes of appointments)’*  Ca: *'It is also important for me to go to the [clinical centre]. We had thought, since my husband and I had to attend the parent training together: "It's Friday night, let's leave the children with their grandmother and carve out some time before or after for the two of us". […] I liked the idea of ​​being with my husband, attending parent training with my husband, meeting other people and having time or an evening for ourselves’* |
| *Usability of contents* |  |
| Usability of brief wellness activity | Ca: *‘Before starting the meeting I had to turn on the computer, leave my child with the carer, I felt a little agitated and the breathing activity really helped me to relax’*  Ca: *‘I personally did not find the breathing exercise very useful because I could not relax with my eyes closed’* |
| Clarity of videorecorded modelling of strategies | Ca: *‘The videorecorded modelling of strategies in my opinion was what collected everything that was said, it was very easy for us to practice at home after watching the video’* |
| Low realism of videorecorded modelling of strategies | Ca: *‘I found Maya too good, she was too cooperative, she never opposed, she never had tantrums, she was a bit far from our reality because my son is not really like that’* |
| Tailoring of strategies | Ca: *‘it was structured on the basis of our child… the interventionists were getting better and better acquainted with our children, they were able to give advice and personalized suggestions’* |
| *Perceived clinical outcomes* |  |
| Not feeling lonely | Ca: *‘Knowing that other mothers have difficulties with their children makes me feel that I am not alone with this difficulty, that this does not happen only with my child’* |
| Self-efficacy | Ca: *‘I have learned a lot and I am more confident in putting these strategies into practice’*  Ca: *‘The tools you gave me give me a little more self-confidence to deal with situations’* |
| Empowerment | Ca: *‘It taught me to think - it seems strange - to think first about what I'm doing, what I'm proposing to my son. It is difficult because some things become less instinctive […] thinking about how to propose a play routine in a structured way is a little less instinctive, but it’s a very important thing’*  Ca*: ‘This experience made me reflect on many things, even on myself, maybe I don't have to be the one to do everything, I always do everything myself and sometimes I exclude my husband, but I understood that he too must participate and that I have to take my space’*  Ca: *‘It was a way to get to know myself too, that’s why I say that if I improve as a person, my son also improves. If I am in a good mood, my son who is my mirror does the same things’* |
| Acquisition of skills | Ca*: ‘I learned a lot, before the course I didn't know how to do what I do now with my son. I think there are mistakes, but they are few, I know I need to improve’*  Ca: *‘I understood how to manage things that previously seemed unmanageable to me’* |
